# Community Structure of Aquatic Insects Adapted to Lentic Water Environments, and Fine-Scale Analyses of Local Population Structures and the Genetic Structures of an Endangered Giant Water Bug *Appasus japonicus*

**DOI:** 10.3390/insects11060389

**Published:** 2020-06-23

**Authors:** Kazuhiro Tomita, Tomoya Suzuki, Koki Yano, Koji Tojo

**Affiliations:** 1 Department of Biology, Faculty of Science, Shinshu University, Matsumoto 390-8621, Japan; kazuhiro.tomita920@gmail.com (K.T.); suzuki_t@shinshu-u.ac.jp (T.S.); 2 Division of Mountain and Environmental Science, Interdisciplinary Graduate School of Medicine, Science and Technology, Shinshu University, Matsumoto 390-8621, Japan; 18hs406b@shinshu-u.ac.jp; 3 Institute of Mountain Science, Shinshu University, Matsumoto 390-8621, Japan

**Keywords:** aquatic insects, biodiversity, DNA, genetic diversity, pond, source−sink relationship, species diversity

## Abstract

Environments such as floodplains and the marshlands of rivers, lakes and ponds, are important habitats for aquatic insects adapted to lentic water conditions. In addition, ponds and paddy fields artificially created for agriculture are also important alternative habitats for lotic water-dependent wildlife. In this study, we focused on aquatic insects in ponds in the Matsumoto Basin, located in the center of Japan. Although this is an urbanized area, aquatic animals adapted to floodplains inhabit it at a relatively high density for Japan. We conducted a multifaceted evaluation of the environments of the 33 ponds in this region and conducted a survey of the aquatic insect fauna inhabiting them. In this study, we conducted quantitative sampling, focusing on two insect orders adapted to large-scale lentic water environments (i.e., Heteroptera and Coleoptera), and observed five species of three families and 16 species of five families from the Matsumoto Basin, respectively. Within these species, eight endangered species were included. Furthermore, we carried out a genetic structure analysis for the giant water bug, *Appasus japonicus*, inhabiting these ponds in high density, and conducted a comparative evaluation of their genetic diversity between these ponds. A total of 530 specimens of *A. japonicus* were genetically analyzed for the mitochondrial DNA COI region, and 26 haplotypes were observed. The degree of genetic diversity between the ponds was clearly demonstrated. In addition, we discussed the wintering possibilities for the giant water bugs based on their corresponding surrounding environmental factors, and comprehensively discussed their “source−sink” relationships in this region. Therefore, this is a comprehensive study focused on the relevant environmental factors, diversification of their community structures, their population structures, and their genetic structure at a fine scale.

## 1. Introduction

Environments such as floodplains and the marshes of rivers, lakes, and ponds, are important habitats for aquatic insects adapted to lentic water conditions [1,2,3]. Such lentic water environments are very important not only for aquatic insects adapted to lentic water conditions, but also for many aquatic species [4,5], and even for species adapted to lotic water conditions. This is because such environments function as areas of refuge during floods, overwintering areas, and as breeding areas [6,7]. However, in recent years, river improvement projects in urban areas have caused a decline in the availability of lentic water environments, such as floodplains and marshland environments, which has become an extremely serious problem. The immobilization of river channels as a result of flood control projects has led to the near-total disappearance of the floodplain environment itself [8,9,10,11].

In the Asian region, for the past several thousand years, the cultivation of rice paddies coinciding with the spread of rice cultivation has served as an alternative habitat to such floodplains [12]. In addition, artificial ponds for agriculture were constructed. In Japan, however, during the past few decades, organisms inhabiting such lentic water areas have become extremely seriously affected by the administration of agricultural chemicals, the deterioration of water quality, and field improvement and development work. In addition, invasive alien organisms have been introduced and become established, which present a large problem. That is, the freshwater lentic environments face quite severe threats, including that of artificial environments [8,9,10].

Under such circumstances, in the recent revision of the Red List of animals, which is routinely conducted by the Ministry of the Environment of the Japanese Government (i.e., the Fourth Red List) [13], a lot of lentic water-adapted aquatic insects have been added to the Red List (especially water beetles and water bugs). When considering conservation measures for such lentic water adapted insects, it is very difficult to preserve all of their habitats. However, it is very important to identify key areas for their conservation, i.e., clarification of hotspots critical to their survival. In addition, it is also extremely important to accumulate a basic biological understanding of each species at the regional population level (e.g., life cycle, reproduction season, and dispersal ability) [14,15]. The relationship between their population structure and their genetic structure is also of great importance [16,17,18]. Therefore, we have conducted this study to analyze the population structure, overwintering success rate, and the genetic structure of the belostomatid giant water bug, *Appasus japonicus* Vuillefroy, 1864, in the Matsumoto Basin area of Nagano Prefecture. This species of giant water bug is one of the Red List’s endangered species. In Japan, almost all species belonging to the same genus and the same family are treated as being endangered. Under such circumstances, *A. japonicus* in this particular study area is the most densely populated habitat in Japan. As such, *A. japonicus* is a typical case of many aquatic insect species adapted to the lentic water environments in our study area.

In this study, we focused on the quality of the environmental conditions in each habitat of *A. japonicus*, and the degree of connectivity or isolation between comparatively closely positioned habitats nearby. Furthermore, we discussed what kind of habitats function as “sources”, and those on the other hand, that function as “sinks”, in the context of the metapopulation. Based on our findings, for the purposes of considering conservation measures, we decided to accumulate a fundamental knowledge base that will enable us to further study what kind of habitat should be targeted for protection and also to indicate more strategic conservation measures. However, as a first step in achieving these goals, it is important to clarify the evolutionary significant units (ESU).

## 2. Materials and Methods

### 2.1. Research Sites and Target Animals

The Matsumoto Basin, which is the designated research area of our study, is a basin surrounded by high altitude mountains of 2000 to 3000 m in all directions. A relatively large number of good quality lentic water environments remain in this area, and it also maintains abundant populations of the giant water bug, *Appasus japonicus* [19,20]. However, in this Matsumoto Basin, two other species of lentic water adapted insects are already treated as being extinct: i.e., *Kirkaldyia deyrolli* (Vuillefroy, 1864) (Heteroptera, Belostomatidae) and *Cybister tripunctatus lateralis* Gschwendtner, 1931 (Coleoptera, Dytiscidae).

**Figure 1:**
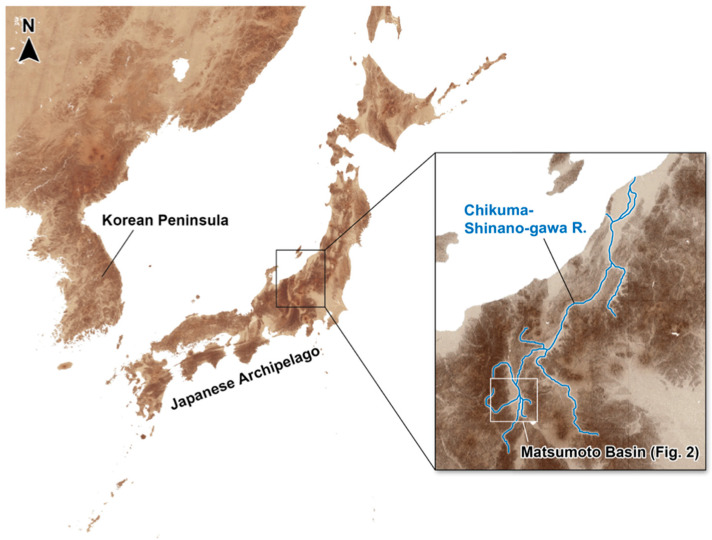
Location of the research area of this study, the Matsumoto Basin.

**Figure 2:**
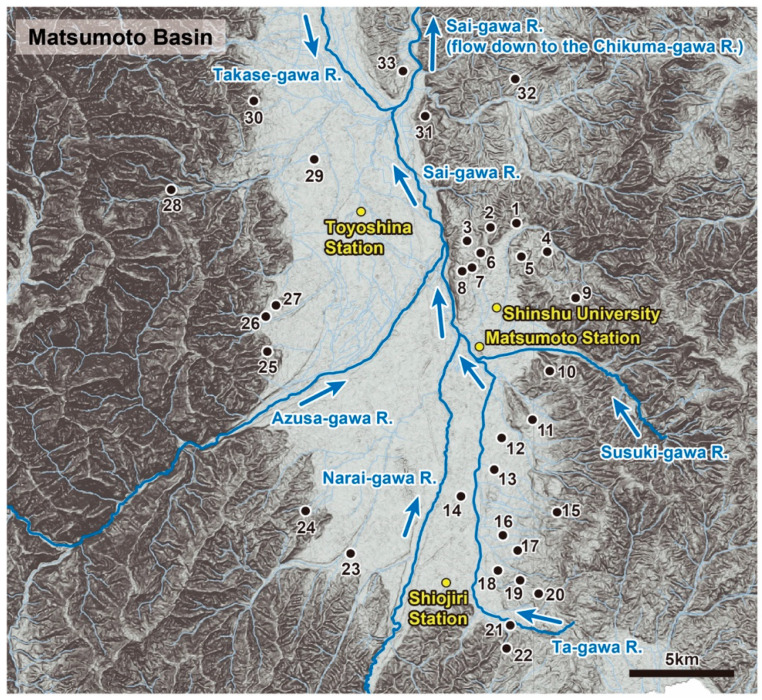
The location of the 33 research sites included in this study within the Matsumoto Basin.

A total of 33 ponds were set as research sites over a wide area almost covering the entire Matsumoto Basin (Table 1; Figure 1 and Figure 2, Supplementary Materials Figure S1). These 33 ponds include a number of types ranging from artificial pond set in concrete to places having a high naturalness. We were not able to include as research sites some ponds located on private properties for which we were unable to obtain permission from the landowners. Regarding population and genetic structure analyses, a preliminary assessment was undertaken collecting a wide variety of insects in the region in order to identify a suitable species as a target of study. From the results of the information gathered, the giant water bug *A. japonicus*, was selected as a target species.

In addition, with respect to other major lentic water adapted aquatic insects collected in the quantitative sampling, species identification was carried out in the field, and the number of each species collected was recorded at the same time. All specimens used in this study, including specimens that have undergone genetic analysis, are stored in the Natural Museum of Shinshu University (SHIN-Z No. 8001-8848).

### 2.2. Measurement of Environmental Factors at Each Pond

We measured thirteen environmental factors as listed below: (1) GPS location (latitude, longitude and altitude), (2) surface area of each pond, (3) perimeter of each pond, (4) percentage of concrete shoreline, (5) percentage of vegetated shoreline, (6) percentage of vegetated total pond surface area, (7) gradient of the pond bed in the zone 3 m from the shoreline (i.e., gradient based on depth measurements taken away from the shore toward the center at three arbitrary points around the pond), (8) opening rate of sky, (9) clarity of pond water, (10) water quality (i.e., concentration of the total nitrogen, T-N; ammonium nitrogen, NH_4_^+^-N or NH_3_^+^-N; phosphate-phosphorous, PO_4_^3−^), (11) number of waterways flowing into the pond, (12) number of waterways flowing out of the pond, and (13) the usage conditions of the surrounding land (e.g., housing, forest, farmland, paddy fields).

The analysis of factors (2) and (3), determination of the shape of each pond researched was based on geographical maps and/or aerial photographs of the Geospatial Information Authority of Japan, and then by analysis using the software Kashmir 3D ver. 9.2.9 [21]. The analysis of factor (5) evaluation was conducted by visually allocating 5 grades. Factor (9) was measured using a self-made transparency meter. Factor (10) was measured using three-pack test kits, WAK-TN-I, WAK-NH_4_ (C), and KR-PO_4_ (Kyoritsu Rika Kenkyu, Tokyo, Japan). Six samples of 100 mL pond water were sampled per pond and stored at 4 °C, and thereafter all samples were moved together a 25 °C room, and then measured in the 25 °C room after standing for 12 h. All of this water quality measurement work was carried out within 3 days from the time of water sampling.

### 2.3. Sampling for Population and Genetic Structure Analyses of *A. japonicus*

Sampling for population and genetic structure analysis was carried out for all of the 33 targeted ponds late in the autumn of 2015. First, the shoreline from which it was possible to perform sampling was equally divided into 6 sections, from which we carried out our quantitative sampling over the predetermined sampling period (i.e., three minutes sampling per zone × 6 zones). We mainly targeted large specimens of two orders (i.e., Heteroptera and Coleoptera), using D-frame sampling nets with a mesh size of 2 mm. Many water striders also inhabited those ponds we surveyed, but we excluded them in preference for the targeted insects, because they escaped frequently from the sampling net during our quantitative survey.

In this survey, when more than 20 individuals of *A. japonicus* were collected, 20 of them were used as genetic analysis specimens. If less than 20 individuals were collected, additional qualitative sampling to obtain the full 20 individuals was carried out. However, in some ponds, we could not eventually collect the 20 required specimens. With respect to other major lentic water adapted aquatic insects collected in the quantitative sampling, species identification was carried out in the field, and the number of each species collected was recorded at the same time. Due to the large proportion of endangered species collected, field identification was performed.

### 2.4. Survey on Overwintering Rate for *A. japonicus*

The same quantitative sampling was conducted in early spring of 2016 for the 29 ponds from which *A. japonicus* specimens were collected in the previous sampling in the late autumn of 2015 (Table S1). Based on these results, we determined the overwintering ponds in which *A. japonicus* was collected in both the late autumn of 2015 and the early spring of 2016. The ratio of the late autumn to the early spring quantitative sampling was regarded as the “overwintering success rate”. However, in calculating the overwintering success rate, only those ponds, from which the number of *A. japonicus* collected in the late autumn research was greater than the median, were included (i.e., >10 specimens were collected in the late autumn of 2015). This overwintering success rate was used for the responsive variable of generalized linear mixed model (GLMM) analysis to reveal a relationship between overwintering success rate and the percentage of concrete shoreline.

### 2.5. Redundancy Analysis (RDA)

Redundancy Analysis (RDA) was conducted to examine the relationship between community structure and environmental factors, using the software R.3.3.2 [22]. Data on the species composition and their individual numbers of lentic water insects collected in each study site were used as the response variables. For the explanatory variables, we used a dataset of 7 environmental factors (altitude, surface area of each pond, percentage of vegetated shoreline, percentage of concrete shoreline, gradient of the pond bed in the zone 3 m from the shoreline, number of waterways flowing into the pond, concentration of the total nitrogen) excluding those that showed little difference among the survey environmental factors and the number of invasive alien species.

### 2.6. Generalized Linear Model (GLM) Analysis

A generalized linear model (GLM) analysis was conducted to examine the relationship between the population structure of each of the detected dominant species and the environmental factors, using the software R.3.3.2 [22]. Since the four species, *A. japonicus*, *Ranatra chinensis* Mayer, 1865, *Ranatra unicolor* Scott, 1874, *Rhantus suturalis* (MacLeay, 1825), were collected from more than half the number of surveyed ponds, and more than 100 individuals in total were collected, respectively (i.e., dominant species), they were subject to this GLM analysis. In addition, as for the species *Laccotrephes japonensis* Scott, 1874, although their collected number was not so large, they were also added to our analysis as they were collected from more than half the number of surveyed ponds. The relative number of collected individuals of each species in our quantitative sampling was used as the response variable. The measured environmental data and the number of collected invasive alien species were used as explanatory variables.

### 2.7. Genetic Analysis

As specimens for genetic analysis (mtDNA COI region) of *A. japonicus*, individuals collected by quantitative sampling, and individuals collected by supplementary qualitative sampling were used. However, although the designated sample of 20 individuals were analyzed properly in 25 ponds, the number collected did not reach 20 individuals for the remaining 8 ponds. The genetic analysis of *A. japonicus*, direct sequencing was performed in accordance with the same method utilized in our previous study [19,20].

Total genomic DNA was extracted from leg tissue, using the DNeasy Blood and Tissue Kit (QIAGEN, Hilden, Germany) according to the manufacturer’s instructions. The total DNA was used for amplifying DNA fragments by polymerase chain reaction (PCR) with a set of universal primers (Folmer et al., 1994): LCO1490 (5′-GGTCAACAAATCATAAAGATATTGG-3′) and HCO2198 (5′-TAAACTTCAGGGTGACCAAAAAATCA-3′). For each reaction, 2.0 µL of 10× (the 10 times concentration) rTaq polymerase buffer, 1.2 µL of 25 mM MgCl_2_, 2.0 µL of 2 mM dNTPmix, 0.1 µL of 5 U/µg rTaq polymerase (TOYOBO, Osaka, Japan), 0.5 µL of each primer, 3.0 µL of extracted DNA and 10.7 µL of SQ were used in 20 µL. The PCR protocol was: 94 °C for 1 min; 35× (35 cycles) (94 °C for 1 min, 50 °C for 1 min, 72 °C for 1 min); 72 °C for 7 min. PCR products were purified using the Microcon Kit (MILLIPORE, Burlington, MA, USA), ExoSAP-IT or ExoSTAR (GE Healthcare, Buckinghamshire, UK). The purified DNA was sequenced directly by an automated method using the DYEnamic ET Terminater Cycle Sequencing Kit (GE Healthcare, Buckinghamshire, UK) or BigDye Terminatior v1.1 Cycle Sequencing Kit (Applied Biosystems, Foster City, CA, USA) on an automated DNA sequencer (an ABI 3130, or ABI 3130xl Genetic Analyzer, Applied Biosystems, Foster City, CA, USA). All of the obtained nucleotide sequence data were registered in GenBank, and their accession numbers are recorded in Table 1.

Phylogenetic analyses were performed using the Bayesian method [23], with MrBayes5D version 3.1.2 [24]; and the maximum likelihood method [25], using RAxML version 8.2.9 [26]. *Appasus major* Esaki 1934, which is a sister species of *A. japonicus*, were used as the outgroup for Bayesian and ML analyses. Nodal support was measured with the posterior probabilities in MrBayes5D, and 1000 bootstrap replicates in RAxML [27]. Prior to the ML and Bayesian phylogenetic estimations, the program Kakusan4 [28] was used to select an appropriate model based on Schwarz’s Bayesian Information Criterion (BIC) [29]. Bayesian analysis used 100 million Markov Chain Monte Carlo (MCMC) cycles with a sampling frequency of 1000. To obtain a consensus tree, data from the initial 10 million cycles were discarded as burn-in.

### 2.8. Analysis between Pairwise Genetic and Geographic Distances

Mantel test was conducted in AIS (Alleles in Space) software [30] to assess the significance of isolation by distance (IBD) between populations with 1000 random permutations on matrices of p-distance values and the geographical distances.

### 2.9. Statistical Analyses Based on the Genetic Data Obtained for *A. japonicus*

Cluster analysis was applied as per the Ward’s method applying the Euclidean distances to assess the similarity of the genetic structures of *A. japonicus* between study sites. The statistical package “mass” by the software R3.3.2 was used [22]. In this calculation, we applied the Chao Index, as one haplotype (H1) showed extremely high dominance [31,32].

In addition, we used nonmetric multidimensional scaling (NMDS) in PRIMER version 6 [33] to assess the similarity of the genetic structure of *A. japonicus* between study sites. We applied a dissimilarity matrix obtained from the Bray-Curtis dissimilarity calculation [34].

## 3. Results

### 3.1. Quantitative Survey of Community Structure among Lentic Water Adapted Aquatic Insect Fauna

The results of our quantitative survey for 33 selected ponds located in the Matsumoto Basin are shown in Figure S1. In this research, only the main lentic water adapted aquatic insects were targeted, and water striders (i.e., Gerridae) were excluded from the survey. Some of the species collected were listed in one of the endangered species categories of the Red List of Japan and/or Nagano Prefecture. Heteropteran aquatic insects were collected more frequently. Among them, the frequencies of the giant water bug, *Appasus japonicus*, and a backswimmer, *Notonecta triguttata* Motschulsky, 1861, being collected, were extremely large. On the other hand, there were ponds from which no aquatic insects targeted in this study were collected (pond No. 13, 14 sites).

In addition, the result confirming the presence or absence of some major invasive alien fauna is also shown in Table S1. In each pond, very few species (i.e., 0–2 species) of invasive alien fauna were observed in this study.

### 3.2. Relationships between the *A. japonicus* Populations and Environmental Factors for the 33 Ponds Studied within the Matsumoto Basin

Table 1 shows the survey results of each environmental factor for the 33 ponds located in the Matsumoto Basin. Table S1 shows the number of individuals collected in the quantitative survey of *A. japonicus*. Specimens of *A. japonicus* were collected from 29 ponds in total, and the maximum number of specimens collected at a given time (18 min: 3 min × 6) was 99 (No. 26), and the minimum number of captures was 0 (No. 13, 14, 27, 28). The GLM analysis for correlations between the numbers captured of *A. japonicus* and the corresponding environmental factors showed significant correlation with the following five factors: (2) surface area of each pond, (4) percentage of concrete shoreline, (5) percentage of vegetated shoreline, (6) percentage of vegetated total pond surface area, (7) gradient of the pond bed in the zone 3 m from the shoreline, (12) number of waterways flowing out of the pond.

### 3.3. Survey on the Successful Overwintering Rate of the Giant Water Bug *A. japonicus*

The quantities collected of the giant water bug, *A. japonicus*, in the quantitative surveys of the autumn of 2015 and early spring of 2016, are shown in Table S1. The highest overwintering rate was observed at the No. 21 pond site (55.0%, 11/20). Thereafter in descending order there was; 41.7% (10/24) at pond No. 24, 35.7% (10/28) at pond No. 9, 33.8% (27/80) at pond No. 16, and 31.8% (7/22) at pond No. 22. Of the others, several ponds with a very low or nil overwintering rate were also confirmed including: site No. 3 (1/10), No. 5 (0/2), No. 7 (0/10), No. 23 (0/1), and No. 32 (0/4). Furthermore, several ponds also showed that although a comparatively large number of specimens were collected in the autumn of 2015, those numbers had drastically decreased by early spring of 2016: e.g., 2.2% (1/44) at No. 31 pond site, 5.9% (2/34) at the No. 11 pond, and 9.5% (2/21) at the No. 6 pond.

### 3.4. Results of Genetic Analysis of *A. japonicus*

**Figure 3:**
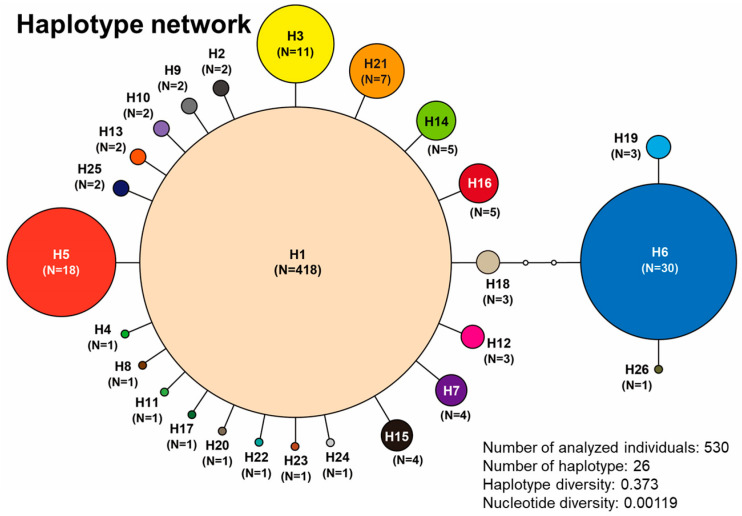
Network of the observed haplotypes of *Appasus japonicus* within the Matsumoto Basin. The sizes of the circles are proportional to the frequency of the haplotype to which they belong, while the small hollow circles represent unobserved haplotypes.

**Figure 4:**
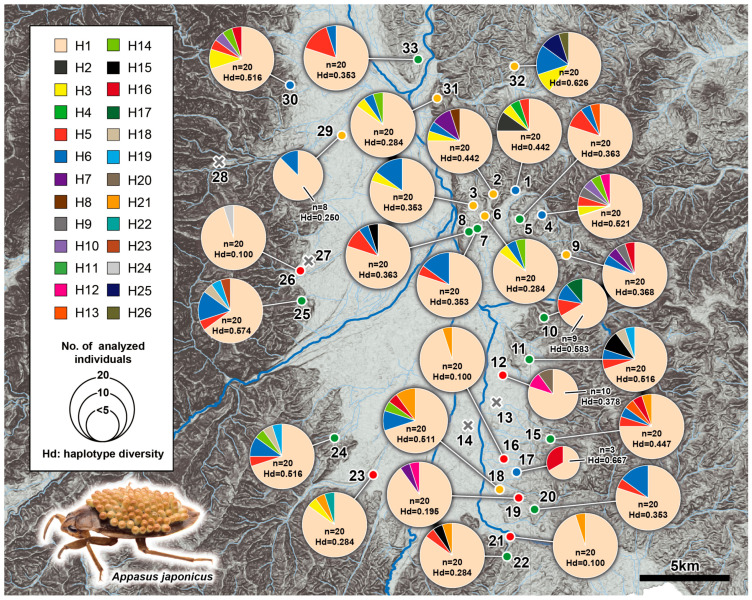
The haplotypes observed at each survey site and their composition ratio.

From the 29 ponds where the specimens of *A. japonicus* were collected, 20 individuals were arbitrarily extracted from those specimens collected at each pond and genetically analyzed. With respect to the ponds where the number of captures was less than 20 individuals, as many of the specimens as possible were analyzed. Genetic analysis (mtDNA COI region) was performed on a total of 530 *A. japonicus* specimens, and 26 haplotypes were observed. The relationships between all observed 26 haplotypes are shown in Figure 3. The most dominant haplotype (i.e., H1 haplotype) was the overwhelming majority of specimens collected, and it was observed in all 29 ponds examined. The second and third most dominant haplotypes (H6 and H5 haplotypes) were also observed in many of the ponds (Table 2, Figure 3). The majority of haplotypes have a single step base substitution relationship from the dominant H1 haplotype, and they all had a substitution relationship of, at the most, 6 steps (Figure 3). The composition of the haplotypes observed in each pond, and the results of haplotype and nucleotide diversity analysis at the intra-pond level are shown in Figure 4. The haplotype diversity (Hd) of *A. japonicus* over the entire Matsumoto Basin was 0.373, and the nucleotide diversity (Pi) was 0.00119. The site numbers of the ponds in which high genetic diversity was observed are listed as follows (ponds for which the genetically analyzed sample number was less than 20 were excluded): No. 4 (Hd = 0.521, Pi = 0.01813), No. 11 (Hd = 0.516, Pi = 0.00186), No. 24 (Hd = 0.516, Pi = 0.00219), No. 25 (Hd = 0.574, Pi = 0.00259), No. 29 (Hd = 0.516, Pi = 0.00152), and No. 32 (Hd = 0.626, Pi = 0.00278).

**Figure 5:**
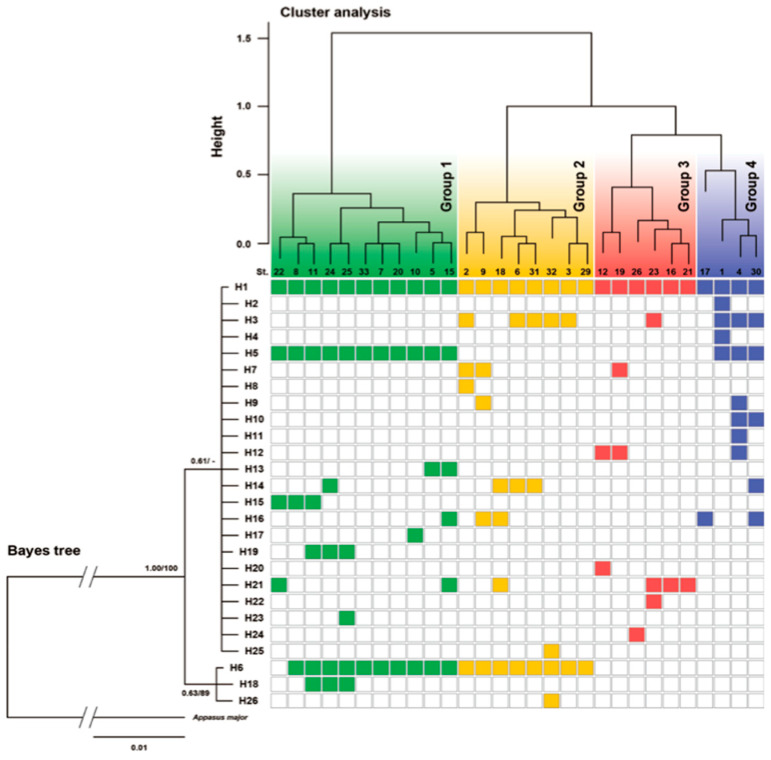
Dendrogram based on an agglomerate hierarchical clustering (Ward’s method) for the 29 research sites analyzed (upper part), and the phylogenetic tree of the Bayesian analysis from nucleotide sequences of all of the observed haplotypes (left part). Cluster analysis: representation of the genetic structure of groups of *Appasus japonicus* according to their genetic data obtained from the mtDNA COI region. Bayesian tree: the tree shape shows the result of a Bayesian analysis, and the numerical value of each node shows the Bayesian posterior probability and their ML bootstrap value (Bayes/ML). Colored squares indicate the ponds in which the corresponding haplotype was detected.

**Figure 6:**
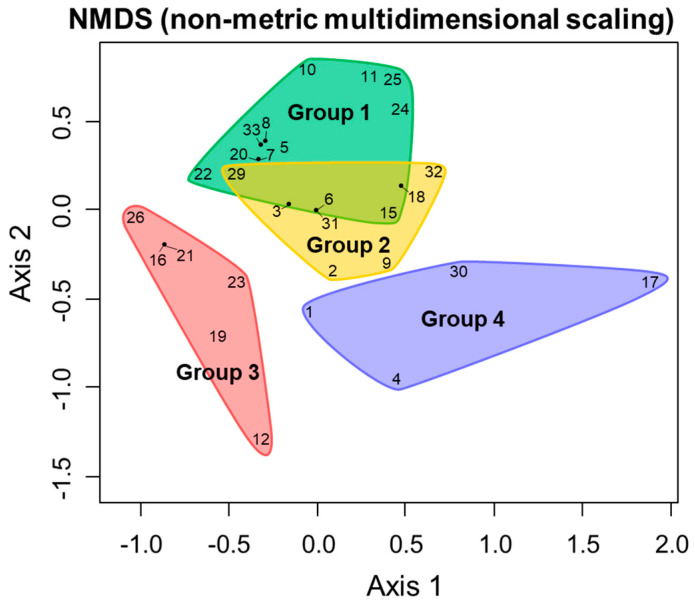
Non-metric multidimensional scaling (NMDS) plots for the data sets of each configuration pattern, for the detected haplotypes based on the mitochondrial DNA COI region observed at each site. The enclosed range and color scheme of each plot corresponds to the group divisions and color scheme of the cluster analysis shown in Figure 5.

**Figure 7:**
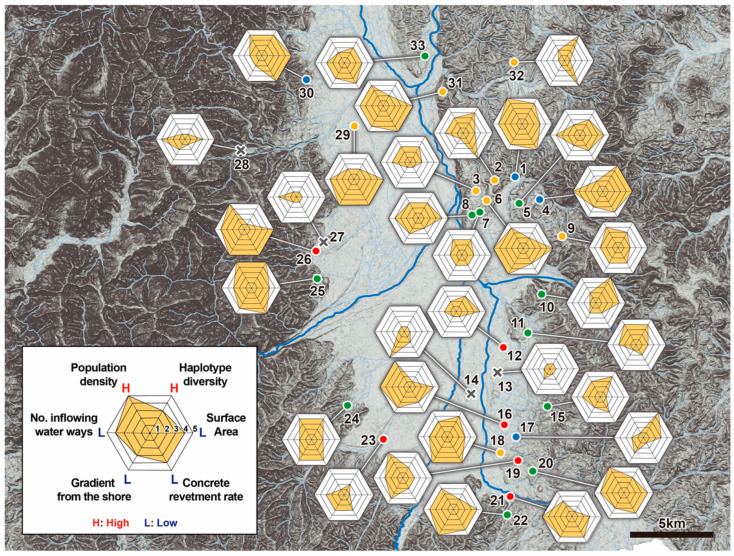
Radar chart of each research pond showing the following environmental factors: population density and genetic diversity of *Appasus japonicus* vs. environmental factors at each pond (i.e., surface area, concrete revetment rate, gradient from the shore, number of inflowing waterways). Regarding the five grades of evaluation of each factor, the median value of each evaluated factor was given the grade of three, with a maximum evaluation of the 5th grade, and a minimum evaluation of the 1st grade. That is, the larger the area and the more well-balanced the chart, the more optimal the environmental conditions for maintaining *A. japonicus* populations and their genetic diversity.

Based on the results of these genetic analyses, to obtain the comparative genetic structures at the intra-pond level, a cluster analysis and an NMDS analysis were conducted, the results of which are shown in Figure 5 and Figure 6. The result of the cluster analysis was largely divided into four clusters (Figure 5). The respective geographical distribution of their ponds, constituting these four genetic clusters, is shown in Figure 4 and Figure 7. It was observed that the geographically close ponds did not necessarily have a correspondingly similar genetic structure. On the other hand, NMDS analysis using the same data set showed a slightly different result from the cluster analysis (Figure 6). Although the groups with the yellow and green backgrounds, were positioned as being different clusters from each other in the cluster analysis, they were shown to be almost the same group in this NMDS analysis. Regarding the remaining two clusters indicated by their red and blue background colors, their distinct grouping was also supported by the NMDS analysis.

### 3.5. Comprehensive Evaluation of Population Structure and Genetic Structure of *A. japonicus*, and Environmental Factors

Figure 7 shows a radar chart showing the relationships between the environmental factors, which are suggested to correlate with the population structure, and genetic diversity of the *A. japonicus* populations. For the purposes of this study, each environmental factor was evaluated as being one of five grades. Although it is also described in the legend of Figure 7, the median value of each evaluated factor was given the grade of three. Then, the radar chart was prepared using the five grades of evaluation, with a maximum evaluation of the 5th grade, and a minimum evaluation of the 1st grade. That is, the larger the area and the more well-balanced the chart, the more optimal the environmental conditions for maintaining *A. japonicus* populations and their genetic diversity (e.g., pond No. 1, 18, 20, 22, 25 and 31 sites).

### 3.6. Survey on the Successful Overwintering Rate of the Giant Water Bug *A. japonicus*

The quantities collected of the giant water bug, *A. japonicus*, in the quantitative surveys of the autumn of 2015 and early spring of 2016, are shown in Table S1. The highest overwintering rate was observed at the No. 21 pond site (55.0%, 11/20). Thereafter in descending order there was; 41.7% (10/24) at pond No. 24, 35.7% (10/28) at pond No. 9, 33.8% (27/80) at pond No. 16, and 31.8% (7/22) at pond No. 22. Other taken those, several ponds with a very low or nil overwintering rate were also confirmed including: Site No. 3 (1/10), No.5 (0/2), No. 7 (0/10), No. 23 (0/1), and No. 32 (0/4). Furthermore, several ponds also showed that although a comparatively large number of specimens were collected in the Autumn of 2015, that number had drastically decreased by early spring of 2016: e.g., 2.2% (1/44) at No. 31 pond site, 5.9% (2/34) at the No. 11 pond, and 9.5% (2/21) at the No. 6 pond. 

**Figure 8:**
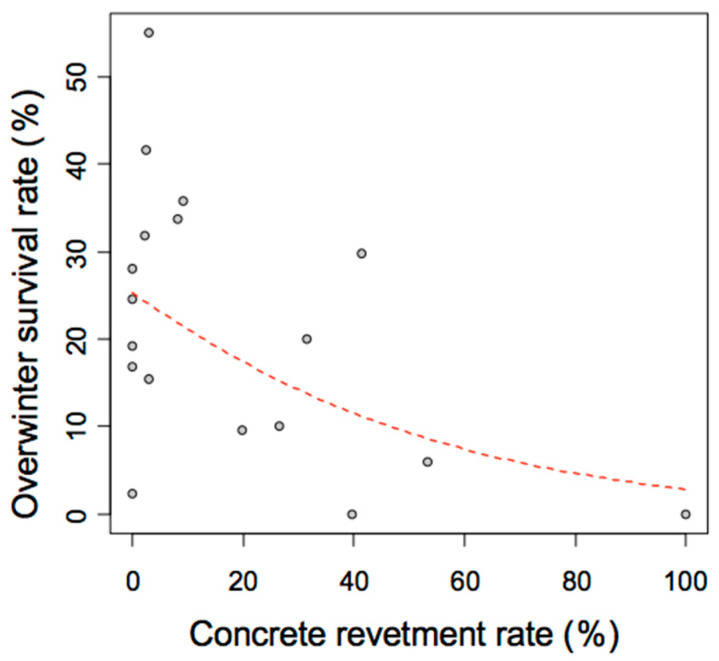
Relationship between overwintering survival rate of *Appasus japonicus* vs. the rate of concrete revetment at each pond (estimated by GLMM: *p* < 0.05).

The most important requirement for ponds to function as sources is for the aquatic insects can successfully overwinter. In this study on overwintering of *A. japonicus*, it was found that the overwintering success rate varies greatly depending on the target ponds. In addition, it was clearly shown that the success rate of wintering has a strong negative correlation with the concrete revetment rate (Figure 8).

## 4. Discussion

### 4.1. Environmental Factors Affecting the Community and Population Structure of Aquatic Insects

In this study we targeted 33 ponds in a single basin, and compared the pond environments, the fauna and species diversity of aquatic insects, and the genetic structure and genetic diversity of a giant water bug, *Appasus japonicus* (Table 1 and Table 2, Table S1, Figure 1, Figure 2, Figure 3, Figure 4, Figure 5, Figure 6, Figure 7 and Figure 8, Figures S1 and S2). This is the first case study in which a detailed fine-scale survey has been conducted on aquatic insects that inhabit ponds in Japan. Although in a previous study no genetic analysis was conducted, it did investigate the effects of various environmental factors in irrigation ponds on their lentic aquatic insect community structures, using the “Canonical Correspondence Analysis (CCA)” method [35]. In the previous study, the species richness of emergent plants in irrigation ponds was evaluated as being the highest contributing factor towards the community structure of the lentic aquatic insects present. The Spearman’s rank correlation coefficient test reported a negative correlation between the number of aquatic insects and the percentage of concreted shoreline, and a positive correlation to the total percentage of vegetated pond surface area [35]. In the previous study, the order Odonata (i.e., dragonflies and damselflies) was included for targets with heteropteran and coleopteran aquatic insects. Although the odonatan species accounted for an especially large proportion in the community, it was a somewhat different study target to the lentic water insect community targeted in our research. However, the pattern of a high correlation between the percentages of concrete shoreline to the corresponding community structures of aquatic insects showed agreement between the previous study and our study. In addition, the result of the “Redundancy Analysis (RDA)” in this study indicated that the gradient of the pond bed to the shoreline was the environmental factor having the largest contribution toward the community structure of the corresponding lentic water inhabiting insects. Therefore, it is generally suggested that the shoreline environments and vegetation play an extremely important role in terms of the species diversity of lentic aquatic insects.

On the other hand, in our study of water scorpions, *Laccotrephes japonensis*, more than expected were captured in those ponds where the percentage of concrete shoreline was somewhat higher (ca. 60%). This trend was also reflected in previous studies. In such cases of ponds having a high percentage of concrete shoreline as described above, where overhanging land-based plants reached the water surface, water scorpions were collected in such microhabitats. The percentage of vegetated shoreline and vegetated total pond surface area measured in this study included cases where overhanging land plants reached the water surface, and it covered not only herbaceous plants but also woody plants. In our study, since water scorpions were collected from 19 ponds (ca. 58%), they may have originated from several robust habitats that exist abundantly in the surroundings.

### 4.2. Environmental Factors That Influence the Intra-Specific Structure in Several Dominant Species of Aquatic Insects

As for the dominant aquatic insect species (i.e., the belostomatid water bugs, *A. japonicus*, *Ranatra chinensis*, *Ranatra unicolor*, *Rhantus suturalis* and *L. japonensis*), and with respect to the environmental factors assessed using the generalized linear model (GLM), it revealed that the relationship between the number of individuals captured relative to the corresponding environmental factors, tended to vary depending on the species.

The analyses of the relationships between the community structures of aquatic insects with the environmental factors, were expected to reveal that the shoreline environment was likely to be an extremely important factor for all of the examined species of aquatic insects. However, the significance of the shoreline gradient and/or the percentage of concrete shoreline was recognized in the only two species, i.e., *A. japonicus*, *L. japonensis*. Both of these species are endangered species listed on the “Red List” [13], and so it was suggested that both species are susceptible to environmental changes due to artificial modification of the shoreline environments. Especially for A. japonicus, concrete revetment decrease the overwintering success rate (Figure 8).

The interspecific differences with regard to the influence of the surrounding environments observed for each species in this study may reflect the differences in their dispersal capability (i.e., *A. japonicus* and *L. japonensis* vs. *R. chinensis* and *R. unicolor*). *Appasus japonicus* and *L. japonensis* have a low dispersal ability compared to the *Ranatra* species, and their distribution pattern tends to be more isolated and scattered. It is thought that they are more susceptible to artificial influences in their habitat environments. On the other hand, the remaining two species of the genus *Ranatra* have a relatively high dispersal ability, whereby it is possible to move between various water bodies, and they are thought less likely to be affected by changes in their habitat.

Among the four heteropteran water bugs which were collected in this study, only one species, *R. unicolor* exhibited patterns of environmental preference for each environmental factor contrary to the other three species. In addition, only within *A. japonicus*, was significance observed between the population density and the number of inflowing water sources. As the number of waterways flowing into the pond increased, the population density tended to decrease (Table 1 and Table S1). In such cases where the ponds are located in mountainous areas, it may be caused by the decreased temperature of the ponds due to the inflow of the cold mountain water. It was reported that the developmental speed of the *Appasus* bugs was strongly influenced by water temperature [36]. Unfortunately, in this study, we did not collect water temperature variance data between the surveyed ponds, and so we cannot discuss this matter more deeply than this. However, such differences in the degree of influence of each environmental factor may suggest basic patterns of habitat differentiation (i.e., some niche differentiation) among these water bug species.

Regarding the relationships with other species groups (i.e., the interspecific interaction), only in *Rhantus* beetles (Colymbetinae) was a relationship observed whereby the number of individuals collected decreased significantly when the number of invasive alien species collected together at the same location increased (Figure S1). Among all the species studied, although they are all prey to other species, since the targeted four heteropteran bugs are less mobile than the *Rhantus* beetles, they are less likely to be found by predators, so they are less susceptible to invasive alien species rather than the *Rhantus*. Thus it is thought that it is less likely for the four heteropterans to be affected by predation pressure.

### 4.3. Characteristics of Genetic Structure Analysis of *A. japonicus*

All 26 haplotypes observed in our genetic structure analysis of *A. japonicus* in the Matsumoto Basin were grouped in “Clade I” among the three intraspecific lineages known so far [20]. This is an expected result when considering the regional nature of the Matsumoto Basin. However, it was interesting that so many haplotypes were observed within a single basin (Table 2, Figure 4 and Figure 5). Therefore, we think that the Matsumoto Basin populations constitute a robust regional group, in a habitat which is extremely well-suited to *A. japonicus*. In view of the situation wherein extinction is common across wide areas of the Japanese Archipelago, there are many lentic water environments in which *A. japonicus* thrives in considerably high density within the Matsumoto Basin. In fact, even though our survey was carried out to include several ponds with concrete shoreline as a target, the proportion of ponds in which *A. japonicus* could be confirmed was still high (i.e., 29/33 ponds).

Cluster analysis and NMDS analysis based on the genetic structure of *A. japonicus*, whereby ponds were classified into four pond groups, however, ponds within a relatively close range in the Matsumoto Basin did not always belong to the same group (Figure 5, Figure 6 and Figure 7). This is because not only is dispersion occurring between neighboring ponds but also migration between ponds across the entire Matsumoto Basin is occurring, and as a result corresponding gene flow.

However, the result of the haplotype network analysis showed the tendency toward a typical “simultaneous dissipation” pattern, with the core dominant haplotype being the H1 haplotype (Figure 3). That is, in several past glacial periods, it is thought that they underwent a bottleneck situation. Such derived haplotypes from the H1 haplotype, had a relationship in which 22 single base substitutions were detected, indicating a relatively recent divergence era. In addition, *A. japonicus* was found present in many ponds, it seems occasional migration between ponds seem to be occurring. In other words, it is inferred that the entire Matsumoto Basin functions as a typical “Metapopulation” of *A. japonicus* (Figure 4 and Figure S2).

### 4.4. “Source−Sink” Relationships at the Intra-Specific Group Level

Statistical analyses based on environmental factors, population density and genetic diversity of *A. japonicus* were carried out, based on the assumption that they would likely be significant to the source−sink relationships observable within the Matsumoto Basin for *A. japonicus*. First, among 33 targeted ponds, *A. japonicus* were collected in high density in some ponds (i.e., No. 1, 16, 20, 25 and 26 ponds), but not in other ponds by quantitative survey (i.e., No. 13, 14, 27 and 28 ponds; Figure 4 and Figure 7). Furthermore, by adding in the viewpoint of genetic diversity, some ponds with high density and high genetic diversity, that is, with high robustness were found (i.e., No. 1 and 25 ponds; Figure 4 and Figure 7). It was considered that such particular ponds in the Matsumoto Basin, which were judged to be particularly excellent habitats, took on the function as “sources” in the “Metapopulation” system, and were the hub of movement and dispersion in the horizontal direction.

On the other hand, there were several ponds with a large decrease in density the following spring, despite many *A. japonicus* inhabitants the previous autumn (i.e., No. 3, 6, 15 and 31 ponds). These ponds are considered “sinks”, like the low-density ponds originally (ponds with low overwintering success rate are likely to be typical “sink” ponds; Figure 7 and Figure 8). These are considered temporary habitats used in the limited seasons from late spring to autumn. Therefore, within the fine-scaled geographical area of the Matsumoto Basin, many lentic habitats of *A. japonicus* connected by the “source−sink” relationships are close to each other, and the “Metapopulation” of the entire region is maintained well.

## 5. Conclusions

In this study, a quantitative survey of aquatic insects was carried out in lentic environments (i.e., total of 33 ponds) in the Matsumoto Basin located in the center of the Japanese Archipelago. In addition, a supplementary qualitative survey was also conducted. Various environmental factors were measured in all ponds. As a result of this, we observed five species of three families of Heteroptera and 16 species of five families of Coleoptera, including eight endangered species. Furthermore, we carried out a genetic structure analysis for the giant water bug, *Appasus japonicus*, inhabiting these ponds in high density and conducted a comparative evaluation of their genetic diversity between these ponds. A total of 530 specimens of *A. japonicus* were genetically analyzed for the mitochondrial DNA COI region, and 26 haplotypes were observed. The degree of genetic diversity between the ponds was clearly demonstrated. In addition, we discussed the overwintering possibilities for the giant water bugs, based on their corresponding surrounding environmental factors, and comprehensively discussed their “source−sink” relationships in this region. Therefore, this is a comprehensive study focused on the relevant environmental factors, diversification of their community structures, their population structures, and their genetic structure at a fine scale. The clarity of source−sink relationships is very important in terms of the assessment of important bodies of water for the conservation of *A. japonicus*, which is also an endangered species. In particular, conservation of this carnivorous aquatic insect, also contributes to conservation of the ecological balance with respect to the many benthic species living under their “umbrella” within their robust habitat environments.

## Figures and Tables

**Table 1:** Geographical information on the research sites and measurement results of each environmental factor.

Site No.	Locality	City/Village	Latitude (N)	Longitude (E)	Altitude (m)	Surface (m^2^)	Perimeter Length (m)	Vegetation Rate *^1^	Shoreline Vegetation Rate (%)	Concrete Revetment Rate (%)	Gradient from the Shore Mean ± SD (%)			Inflow the Number of Waterways	Outflow the Number of Waterways	Water Transparency Mean ± SD (cm)			TN Mean ± SD (mg/L)			NH_4_^+^-N Mean ± SD (mg/L)			PO_4_^3−^-P Mean ± SD (mg/L)			Genbank Accession No.
1	Shinagura	Matsumoto	36.2923	137.9904	828	940	121	III	66.7	0.0	33.7	±	15.8	1	1	46.4	±	6.8	0.0	±	0.0	0.0	±	0.0	0.13	±	4.71 × 10^−2^	LC556393–LC556412
2	Okada-ibuka	Matsumoto	36.2897	137.9742	776	2470	206	II	100.0	0.0	66.2	±	29.7	2	1	36.2	±	3.9	1.7	±	2.4	0.0	±	0.0	0.10	±	1.39 × 10^−17^	LC556413–LC556432
3	Shimauchi	Matsumoto	36.2827	137.9602	848	3470	231	II	12.2	100.0	61.5	±	17.0	2	1	14.8	±	0.8	0.0	±	0.0	0.0	±	0.0	0.10	±	1.39 × 10^−17^	LC556433–LC556452
4	Misayama	Matsumoto	36.2778	138.0091	846	180	55	II	57.4	5.2	42.4	±	16.2	0	1	49.3	±	3.5	5.0	±	0.0	0.0	±	0.0	0.10	±	1.39 × 10^−17^	LC556453–LC556472
5	Hora	Matsumoto	36.2755	137.9929	802	590	95	II	49.2	10.8	46.3	±	12.5	1	1	68.8	±	20.2	0.0	±	0.0	0.0	±	0.0	0.10	±	1.39 × 10^−17^	LC556473–LC556492
6	Okada-ibuka	Matsumoto	36.2768	137.9685	767	120	46	II	48.5	19.8	22.5	±	13.8	0	0	30.7	±	0.4	0.0	±	0.0	0.0	±	0.0	0.10	±	1.39 × 10^−17^	LC556493–LC556512
7	Okada-shimookada	Matsumoto	36.2704	137.9630	736	2270	188	II	17.4	39.6	48.8	±	20.3	3	1	32.4	±	0.7	0.0	±	0.0	0.0	±	0.0	0.10	±	1.39 × 10^−17^	LC556513–LC556532
8	Okada-shimookada	Matsumoto	36.2679	137.9578	737	650	98	II	42.5	31.5	44.5	±	29.3	1	1	56.9	±	5.8	0.0	±	0.0	0.0	±	0.0	0.13	±	4.71 × 10^−2^	LC556533–LC556552
9	Satoyamabe	Matsumoto	36.2545	138.0268	1205	3520	237	II	57.2	9.2	42.0	±	25.0	2	1	53.1	±	9.0	1.7	±	2.4	0.0	±	0.0	0.10	±	1.39 × 10^−17^	LC556553–LC556572
10	Satoyamabe	Matsumoto	36.2183	138.0109	835	810	120	II	31.5	39.8	54.3	±	26.9	4	1	>100.0			3.3	±	2.4	0.0	±	0.0	0.10	±	1.39 × 10^−17^	LC556573–LC556581
11	Nakayama	Matsumoto	36.1941	138.0004	726	2540	215	V	48.4	53.2	67.5	±	26.7	2	1	83.1	±	10.2	1.7	±	2.4	0.0	±	0.0	0.10	±	1.39 × 10^−17^	LC556582–LC556601
12	Kotobuki-toyooka	Matsumoto	36.1857	137.9816	646	670	106	II	66.2	72.8	56.8	±	29.3	3	1	66.8	±	6.6	6.7	±	2.4	0.0	±	0.0	0.10	±	1.39 × 10^−17^	LC556602–LC556611
13	Kotobuki-koaka	Matsumoto	36.1695	137.9769	661	4840	268	II	40.9	59.1	47.0	±	19.9	4	1	37.9	±	6.6	0.0	±	0.0	0.0	±	0.0	0.10	±	1.39 × 10^−17^	-
14	Hirooka-yoshida	Shiojiri	36.1564	137.9558	654	5950	333	III	63.4	43.8	24.5	±	14.8	4	1	77.3	±	22.7	0.0	±	0.0	0.0	±	0.0	0.10	±	1.39 × 10^−17^	-
15	Kataoka	Shiojiri	36.1489	138.0155	1030	6000	305	II	43.2	26.6	51.1	±	22.6	2	1	>100.0			0.0	±	0.0	0.0	±	0.0	0.10	±	1.39 × 10^−17^	LC556612–LC556631
16	Kataoka	Shiojiri	36.1374	137.9820	745	540	95	II	47.0	8.1	27.8	±	10.7	0	0	77.2	±	15.3	11.7	±	9.4	1.5	±	0.7	0.10	±	1.39 × 10^−17^	LC556632–LC556651
17	Kataoka	Shiojiri	36.1296	137.9910	813	2020	196	II	47.5	62.3	42.1	±	11.1	3	1	88.4	±	0.4	0.0	±	0.0	0.0	±	0.0	0.10	±	1.39 × 10^−17^	LC556652–LC556654
18	Kataoka	Shiojiri	36.1196	137.9787	734	860	134	II	81.7	0.0	24.3	±	18.4	2	1	>100.0			1.7	±	2.4	0.0	±	0.0	0.10	±	1.39 × 10^−17^	LC556655–LC556674
19	Sajiki	Shiojiri	36.1149	137.9922	799	2600	231	II	61.5	41.3	31.4	±	20.7	2	1	83.7	±	2.5	0.0	±	0.0	0.0	±	0.0	0.10	±	1.39 × 10^−17^	LC556675–LC556694
20	Kakizawa	Shiojiri	36.1078	138.0040	922	620	104	II	71.8	3.0	43.4	±	13.4	1	1	>100.0			1.7	±	2.4	0.0	±	0.0	0.10	±	1.39 × 10^−17^	LC556695–LC556714
21	Kaminishijo	Shiojiri	36.0924	137.9870	781	1600	161	II	97.1	2.9	34.5	±	13.1	0	1	>100.0			0.0	±	0.0	0.0	±	0.0	0.10	±	1.39 × 10^−17^	LC556715–LC556734
22	Kyu-shiojiri	Shiojiri	36.0810	137.9840	916	720	110	II	58.7	2.1	23.3	±	7.9	1	1	>100.0			0.0	±	0.0	0.0	±	0.0	0.10	±	1.39 × 10^−17^	LC556735–LC556754
23	Nishiseba	Asahi	36.1276	137.8889	764	3780	262	II	100.0	21.0	46.2	±	26.0	2	1	>100.0			0.0	±	0.0	0.0	±	0.0	0.10	±	1.39 × 10^−17^	LC556755–LC556774
24	Kami-oike	Yamagata	36.1491	137.8616	799	2130	186	II	65.0	2.3	40.1	±	19.8	3	1	92.3	±	5.7	0.0	±	0.0	0.0	±	0.0	0.10	±	1.39 × 10^−17^	LC556775–LC556794
25	Azusagawa-azusa	Matsumoto	36.2285	137.8381	809	1480	160	II	30.7	0.0	38.3	±	16.7	2	1	>100.0			0.0	±	0.0	0.0	±	0.0	0.10	±	1.39 × 10^−17^	LC556795–LC556814
26	Misato-ogura	Azumino	36.2457	137.8376	744	150	64	III	100.0	0.0	9.0	±	3.4	1	1	>100.0			0.0	±	0.0	0.0	±	0.0	0.10	±	1.39 × 10^−17^	LC556815–LC556834
27	Misato-ogura	Azumino	36.2515	137.8433	742	4860	313	II	13.9	100.0	61.9	±	34.2	2	1	>100.0			0.0	±	0.0	0.0	±	0.0	0.10	±	1.39 × 10^−17^	-
28	Horigane-karasugawa	Azumino	36.3085	137.7797	1178	1100	149	II	14.3	100.0	52.4	±	18.9	1	1	97.3	±	3.8	1.7	±	2.4	0.0	±	0.0	0.10	±	1.39 × 10^−17^	-
29	Hotaka-kashiwabara	Azumino	36.3235	137.8671	585	170	59	II	92.6	0.0	23.7	±	15.8	1	1	17.7	±	2.2	0.0	±	0.0	0.0	±	0.0	0.10	±	1.39 × 10^−17^	LC556835–LC556842
30	Hotaka-ariake	Azumino	36.3525	137.8301	692	120	57	II	38.3	0.0	49.1	±	15.3	3	1	>100.0			0.0	±	0.0	0.0	±	0.0	0.10	±	1.39 × 10^−17^	LC556843–LC556862
31	Akashina-nakagawate	Azumino	36.3448	137.9341	768	110	46	II	100.0	0.0	31.3	±	8.4	1	0	47.0	±	1.3	0.0	±	0.0	0.0	±	0.0	0.10	±	1.39 × 10^−17^	LC556863–LC556882
32	Aida	Matsumoto	36.3633	137.9900	774	7880	502	II	96.1	0.0	64.3	±	37.0	4	2	39.9	±	1.2	0.0	±	0.0	0.0	±	0.0	0.10	±	1.39 × 10^−17^	LC556883–LC556902
33	Akashina-nanaki	Azumino	36.3675	137.9211	546	1100	141	III	72.6	7.6	42.6	±	18.9	2	0	>100.0			0.0	±	0.0	0.0	±	0.0	0.10	±	1.39 × 10^−17^	LC556903–LC556922

*^1^ Regarding the vegetation rate of the whole pond, a graded evaluation was made as follows: I: 0%, II: 1–25%, III: 26–50%, IV: 51–75%, V: 76–99%, VI: 100%.

**Table 2:** Haplotypes observed and genetic diversity at each study site.

Site No.	n	h	Hd	Pi	Haplotype No.																									
					H1	H2	H3	H4	H5	H6	H7	H8	H9	H10	H11	H12	H13	H14	H15	H16	H17	H18	H19	H20	H21	H22	H23	H24	H25	H26
1	20	5	0.442	0.00074	15	2	1	1	1																					
2	20	5	0.442	0.00120	15		1			1	2	1																		
3	20	3	0.353	0.00178	16		1			3																				
4	20	7	0.521	0.01813	14		1		1				1	1	1	1														
5	20	4	0.363	0.00105	16				2	1							1													
6	20	4	0.284	0.00091	17		1			1								1												
7	20	3	0.353	0.00178	16				1	3																				
8	20	4	0.363	0.00105	16				2	1									1											
9	20	5	0.368	0.00106	16					1	1		1							1										
10	9	4	0.583	0.00203	6				1	1											1									
11	20	6	0.516	0.00186	14				1	1									2			1	1							
12	10	3	0.378	0.00061	8											1								1						
15	20	6	0.447	0.00122	15				1	1							1			1					1					
16	20	2	0.100	0.00015	19																				1					
17	3	2	0.667	0.00101	2															1										
18	20	5	0.511	0.00174	14					2								1		1					2					
19	20	3	0.195	0.00030	18						1					1														
20	20	3	0.353	0.00178	16				1	3																				
21	20	2	0.100	0.00015	19																				1					
22	20	4	0.284	0.00046	17				1										1						1					
23	20	4	0.284	0.00046	17		1																		1	1				
24	20	6	0.516	0.00219	14				1	2								1				1	1							
25	20	6	0.574	0.00259	13				1	3												1	1				1			
26	20	2	0.100	0.00015	19																							1		
29	8	2	0.250	0.00152	7					1																				
30	20	6	0.516	0.00090	14		2		1					1				1		1										
31	20	4	0.284	0.00091	17		1			1								1												
32	20	5	0.626	0.00278	12		2			3																			2	1
33	20	3	0.353	0.00102	16				3	1																				
Total	530				418	2	11	1	18	30	4	1	2	2	1	3	2	5	4	5	1	3	3	1	7	1	1	1	2	1

n: number of genetically analyzed specimens; h: number of haplotypes observed; Hd: haplotype diversity; Pi: nucleotide diversity.

## Supplementary Materials

The following are available online at https://www.mdpi.com/2075-4450/11/6/389/s1, Table S1: List of major lentic aquatic insects collected in the quantitative survey (early spring of 2016/autumn of 2015), and the observation of notable invasive alien fauna: observed (+) or unobserved (−). Figure S1: Pictures of all of study sites. Figure S2: Relationships between pairwise genetic distances (uncorrected p-distance) and pairwise geographical distances of the giant water bug, *Appasus japonicus* (530 individuals; correlation of genetic and geographical distances: r = −0.0021520714. Probability of observing a correlation greater than or equal to observed: *p* = 0.5704295704).
