# Supplementary material for: Community Structure of Aquatic Insects Adapted to Lentic Water Environments, and Fine-Scale Analyses of Local Population Structures and the Genetic Structures of an Endangered Giant Water Bug Appasus japonicus
Source: Insects. 2020 Jun 23;11(6):389. doi: 10.3390/insects11060389 (PMC7349394; doi:10.3390/insects11060389)
Supplement: Supplementary file 1 [file insects-11-00389-s001.zip › insects-818721-supp-Figure S1 S2.docx]

**Supplementary Material**


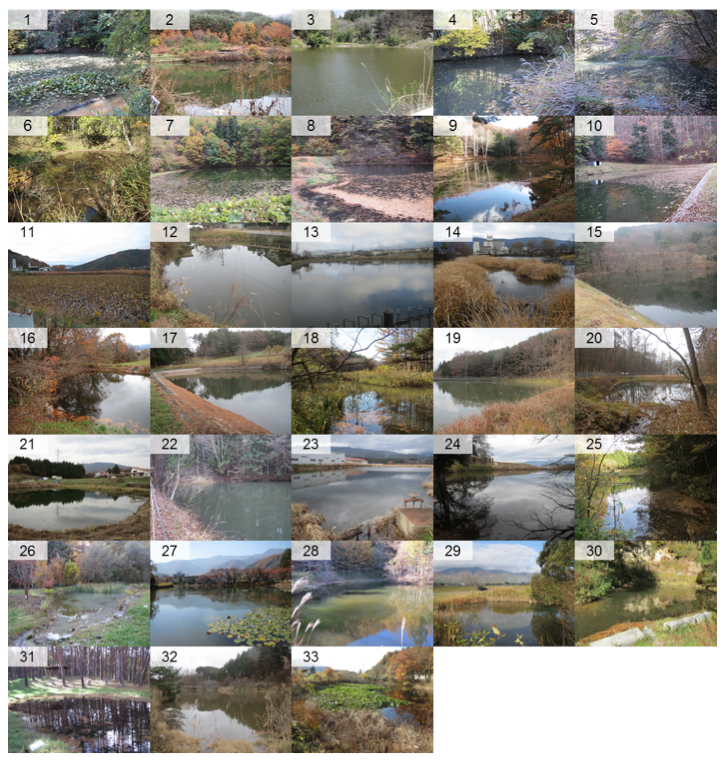


**Figure S1.** Landscapes of all the elected research sites of this study, the Matsumoto Basin.


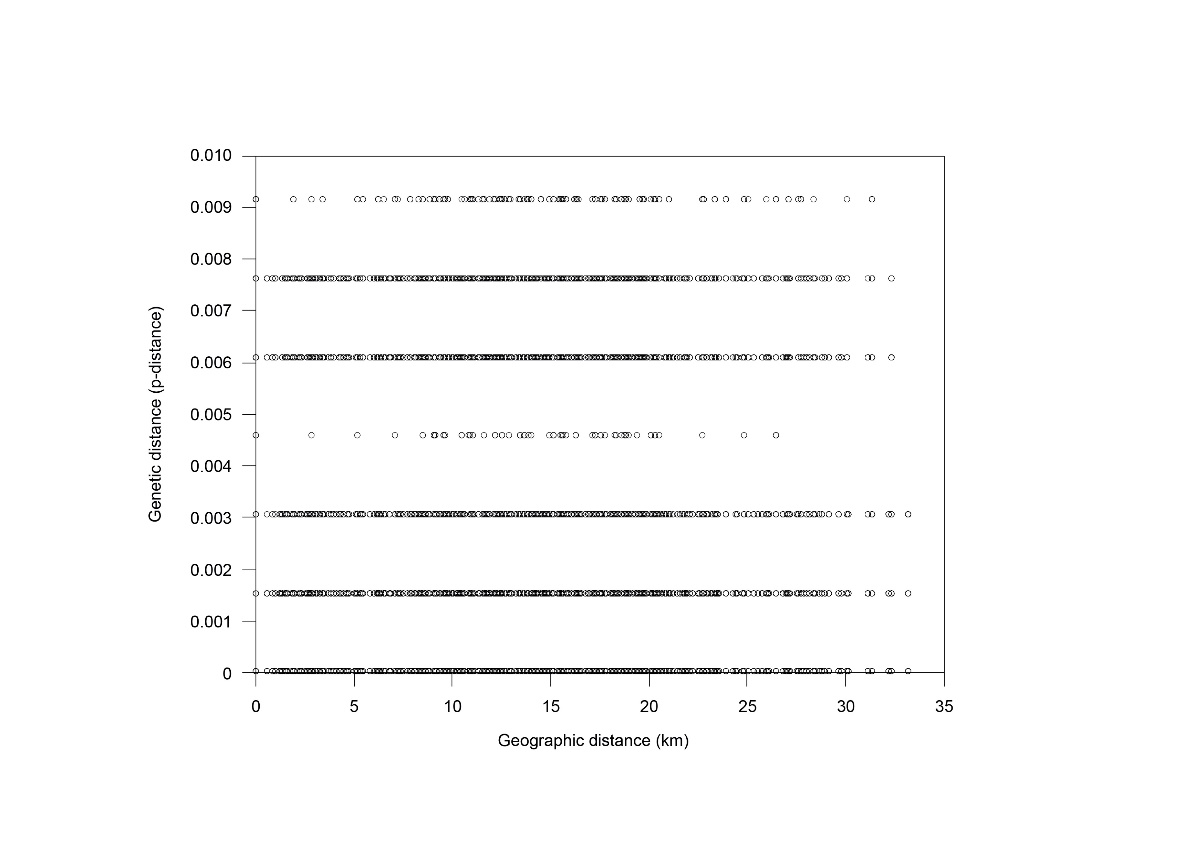


**Figure S2.** Relationships between pairwise genetic distances (uncorrected p-distance) and pairwise geographical distances of the giant water bug, *Appasus japonicus* (530 individuals; Correlation of genetic and geographical distances: r = -0.0021520714. Probability of observing a correlation greater than or equal to observed: P = 0.5704295704).
